# Use of After Action Reports (AARs) to Promote Organizational and Systems Learning in Emergency Preparedness

**DOI:** 10.3390/ijerph9082949

**Published:** 2012-08-16

**Authors:** Elena Savoia, Foluso Agboola, Paul D. Biddinger

**Affiliations:** 1 Department of Biostatistics and Division of Policy Translation and Leadership Development, Harvard School of Public Health, 401 Park Drive, Landmark Center, 3rd Floor East, Boston, MA 02215, USA; 2 Division of Policy Translation and Leadership Development, Harvard School of Public Health, 677 Huntington Avenue, Boston, MA 02115, USA; Email: fagboola@hsph.harvard.edu (F.A.); pbiddinger@partners.org (P.D.B.); 3 Department of Health Policy and Management, Harvard School of Public Health, 677 Huntington Avenue, Boston, MA 02115, USA; 4 Department of Emergency Medicine, Massachusetts General Hospital, Zero Emerson Place, Suite 340, Boston, MA 02114, USA

**Keywords:** lessons learned, after action report (AAR), knowledge management, organizational learning, emergency preparedness

## Abstract

Many public health and healthcare organizations use formal knowledge management practices to identify and disseminate the experiences gained over time. The “lessons-learned” approach is one such example of knowledge management practice applied to the wider concept of organizational learning. In the field of emergency preparedness, the lessons-learned approach stands on the assumption that learning from experience improves practice and minimizes avoidable deaths and negative economic and social consequences of disasters. In this project, we performed a structured review of AARs to analyze how lessons learned from the response to real-incidents may be used to maximize knowledge management and quality improvement practices such as the design of public health emergency preparedness (PHEP) exercises. We chose as a source of data the “Lessons Learned Information Sharing (LLIS.gov)” system, a joined program of the U.S. Department of Homeland Security DHS and FEMA that serves as the national, online repository of lessons learned, best practices, and innovative ideas. We identified recurring challenges reported by various states and local public health agencies in the response to different types of incidents. We also strived to identify the limitations of systematic learning that can be achieved due to existing weaknesses in the way AARs are developed.

## 1. Introduction

In order to improve their performance, many public health and healthcare organizations use formal knowledge management practices to identify and disseminate the experience gained by individuals and groups over time. The “lessons-learned” approach is one such example of knowledge management practice applied to the wider concept of organizational learning. In the field of emergency preparedness, the lessons-learned approach stands on the assumption that learning from experience, whether it be our own experience or others’, and whether it be from real events or simulations, improves practice and minimizes avoidable deaths and negative economic and social consequences of disasters [1,2]. Thus, the appeal of learning from experience to avoid duplicating mistakes is widely appreciated in the emergency preparedness arena, and many organizations have adopted formal procedures for identifying, documenting and disseminating lessons-learned from prior response to emergency situations and simulations. 

Over the years, public health and emergency management agencies have utilized several disparate modes for collecting and sharing such experience, including in-progress reviews, “hot-washes”, various forms of debriefings and after-action reports (AARs) [3]. AARs, originally developed by the U.S. Army, are now widely used by non-military organizations, businesses, and health-care and public health agencies as tools for gathering and documenting evaluations of key processes during the response to both real-incidents and fictional exercises. In fact, in the United States (U.S.), formal AARs are now required by several agencies and organizations that fund, oversee, or regulate aspects of public health and healthcare emergency preparedness and response, including the Assistant Secretary for Preparedness and Response (ASPR), the Centers for Disease Control and Prevention (CDC) and the Joint Commission [4,5]. Yet, despite voluminous attempts to document and learn from prior emergency preparedness system response failures, the challenges experienced in planning and responding to disasters seem to be “learned” over and over again in disaster after disaster [6] suggesting that, while review of incidents and the identification of lessons may be more readily accomplished, true organizational and systems-level learning remains difficult to achieve. Some of this difficulty may have to do with the variability and variety of healthcare and public health “systems” in the U.S. As early as 1988, the Institute of Medicine (IOM) acknowledged that the public health “system” was “a complex network of individuals and organizations that, when working together, can represent ‘what we as a society do collectively to assure the conditions in which people can be healthy’” [7]. However, it has been only recently that greater numbers of researchers have begun to study “public health systems” in earnest. However, beyond the challenges inherent in maintaining disparate and heterogeneous public health systems, Auf der Heide also discussed the common pitfalls seen in the lessons-learned approach itself, commenting that recurring difficulties in responding to disasters are due, in part, to the failure of organizations to produce generalized recommendations that have meaning outside of the context of a specific event [8]. Seid *et al*. suggest that, at the level of individual organizations, this failure relates to a disconnection between the observation of challenges and the implementation of solutions, and advances that information from AARs better drive process change if formalized procedures are developed to follow through improvement efforts [9]. Because of this type of concern, the Federal Emergency Management Agency’s (FEMA) Homeland Security Exercise Evaluation Program (HSEEP) now requires linking of lessons observed to the planned execution of improvement efforts [10]. For example, for each response issue identified in an AAR, HSEEP guidelines recommended that specific work processes and capabilities are identified and targeted to improve organizational or system performance, and exercises and drills used to test the changes being implemented. 

Therefore, there is a need for both a well-ordered and exchangeable information system that compiles post-event reviews of what worked and what did not in the response to specific incidents, as well as a need for structured and rigorous data reviews of aggregations of such reports to help make the most appropriate recommendations for systems-wide changes to current practices. Additionally, it seems only logical that recurring systems-wide challenges observed in AARs data should be incorporated into the structure and content of emergency preparedness exercises to ensure that emergency and disaster simulations regularly and realistically present responders with the most representative and most common response issues that they are likely to face. 

## 2. Study Aims

In this project we have endeavored to perform a structured review of AARs to analyze how lessons learned from the response to real-incidents may be used to maximize knowledge management and quality improvement practices such as the design of public health emergency preparedness (PHEP) exercises for measuring system level preparedness and improvement efforts. We chose as a source of data the “Lessons Learned Information Sharing (LLIS.gov)” system, a joined program of the Department of Homeland Security (DHS) and FEMA that serves as the national, online repository of lessons learned, best practices, and innovative ideas for the emergency management and homeland security communities. A specific section of the LLIS.gov contains a database of AARs submitted by public health agencies, and hospitals describing the response to specific emergencies. 

In this study, we aimed to perform a content analysis of AARs contained in the LLIS.gov database to identify recurring challenges consistently reported by various states and local public health agencies in the response to different types of incidents. We also strived to identify the limitations of systematic learning that can be achieved due to existing weaknesses in the way AARs are developed. With our results, we have attempted to describe how systems can confront these challenges, by ensuring that identified recurring response issues are incorporated into the design of future emergency preparedness exercises.

## 3. Methods

**Figure 1:**
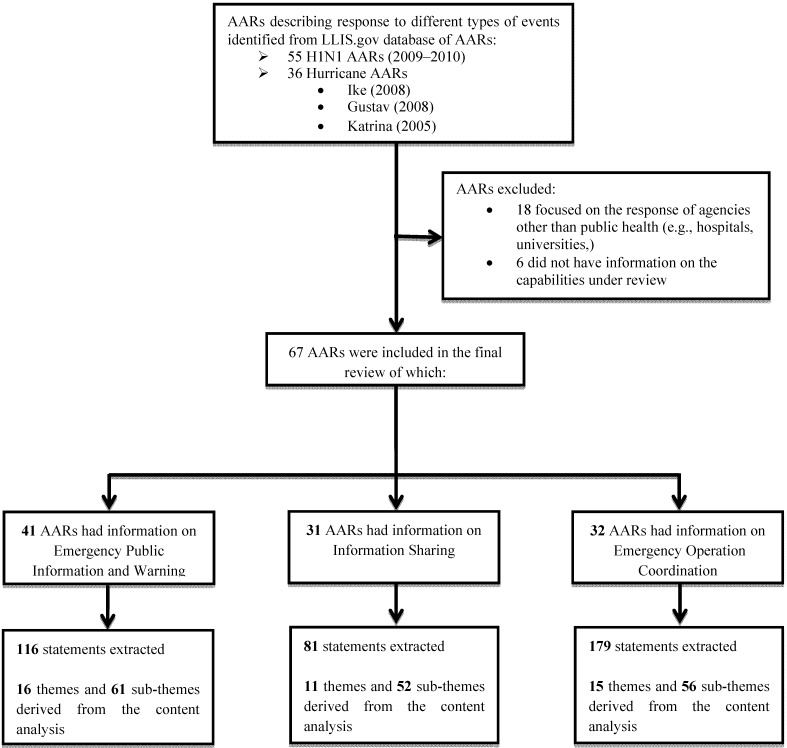
Flowchart showing the AARs review process.

Within the LLIS database we searched for AARs describing the public health system response to two different types of events: the 2009–2010 H1N1 pandemic and three hurricanes Ike (2008), Gustav (2008) and Katrina (2005). AARs were extracted in March 2011 and considered for inclusion in the review if submitted by a U.S. state, county or city public health agency, focused on a public health system response to a real-incidents (as opposed to an exercise), and reported in a semi-structured or structured manner with sufficient information so that analysis of specific public health capabilities could be performed. Each AAR was reviewed, coded and notes taken by two reviewers who independently categorized, sorted, and labeled selected statements within themes and subthemes. Our goal was not only to count the statements, but also to fracture the data and rearrange them into categories that facilitate comparison between events in the same categories [11]. The data abstraction tool that we developed included the following categories: event type, capability(ies) evaluated, challenges faced during the response, and type of format of the AAR. The two reviewers independently identified themes and subthemes that emerged from the text and agreement on such categories was achieved by discussion. See Figure 1 for a description of the reviewing process.

The reviewers analyzed the response issues using the framework provided by the Public Health Preparedness Capabilities developed by U.S. CDC in March 2011 [12]. The analysis focused on the following three capabilities: (1) Emergency Public Information and Warning—*the ability to develop*, *coordinate*, *and disseminate information*, *alerts*, *warnings*, *and notifications to the public and incident management responders*; (2) Information Sharing—*the ability to conduct multijurisdictional, multidisciplinary exchange of health-related information and situational awareness data among federal*, *state*, *local*, *territorial*, *and tribal levels of government, and the private sector*; and (3) Emergency Operations Coordination (EOC)—*the ability to direct and support an event* *or incident with public health or medical implications by establishing a standardized*, *scalable system of oversight*, *organization*, *and supervision consistent with jurisdictional standards and practices and with the National Incident Management System*.

Descriptive statistics were performed to determine the recurrence of the themes by each capability from all AARs (independently of the type of event). Frequencies of subthemes were calculated using the total number of subthemes identified within the pool of statements derived from each theme. Statistical analysis was performed using the statistical package STATA version 11, College Station, Texas. We considered the pool of reviewed AARs as if it were a unified source of information describing the overall response of multiple U.S. public health agencies to the two types of incidents being selected for the analysis. In our analysis, we were not interested in describing differences across agencies which would have been impossible to determine due to the extreme variability in the quality, format and richness of information provided by each AAR. The content analysis of the AARs was performed aiming to identify common themes (response challenges) and subthemes (specific problems within each response challenge) experienced by public health systems across the U.S. in employing such capabilities. 

## 4. Results

Ninety-one AARs met the primary criteria for inclusion in our study. Among the original ninety-one AARs included for evaluation, six did not have any information about the three capabilities being selected, and were therefore excluded, forty-one had sufficient information (at least one statement) for the content analysis of *Emergency Public Information and Warning*, thirty-one for the analysis of *Information Sharing* and thirty-two for the analysis of *Emergency Operations Coordination*. Of the ninety-one AARs, thirty-six focused on the response to hurricanes (twenty-six on Gustav and/or Ike and ten on Katrina) and fifty-five on the response to H1N1. Most were from state health departments (forty-eight), fourteen were from health departments operating at the county level, eleven from health departments serving a city or town, and the rest (eighteen) were from either hospitals, universities, or federal agencies (see Figure 1). 

### 4.1. Emergency Public Information and Warning

Of the forty-one AARs describing a specific challenge associated with the capability of emergency public information and warning, we were able to extrapolate one-hundred-sixteen different statements. Only four of these statements, however, were derived from AARs describing the response to a hurricane, and therefore the results related to the analysis of this specific capability should generally be interpreted in the context of the response to the H1N1 pandemic only. Content analysis of the one-hundred-sixteen statements was performed and sixteen themes and sixty-one sub-themes were derived. Among the sixteen themes the three most frequently reported were: communication difficulties about the vaccine (15%), difficulty in managing the helpline (14%), obstacles in the process of releasing timing information (12%). 

In terms of *communication about the vaccine*, the most frequent issues being reported were related to communication about vaccine safety and rumor control (67%), followed by communication about vaccine distribution-including both quantities and timing of distribution (17.%) and vaccine efficacy (11%). Many public health agencies reported activating a *helpline number* to receive calls from the public, however management issues were frequently consisting of: lack of staff available to cover the service (31%), lack of medical expertise (19%), inadequate publicity of the helpline number (19%), lack of a triage system to re-direct calls (especially those with specific medical content) (12%), and lack of clarity about the triggers for activating and de-activating the service (12%). In terms of *release of timing information*,the most frequent issue was related to the agencies’ ability to process and release new information (50%), which was often reported to be complex and time consuming. As an example, in one AAR reviewed, it was noted by an agency that the formal process for approval and release of information to the public during the emergency was treated the same as it was during routine operation, therefore the need to develop pre-approved messages to hasten the process was suggested. Other issues related to the timing of release of information were either context-specific, such as late evacuation orders, or audience-specific, such as late communication to school districts directors or the media.

A list of most frequent themes and subthemes derived from the analysis of this capability is provided in Table 1. 

**Table 1:** Analysis of themes and sub-themes for the capability of emergency public information and warning.

Emergency Public Information and Warning( *116 statements*)	
Most frequent themes ( proportion *)	Most frequent subthemes within each theme ( proportion **)
Communication difficulties about the vaccine (15%)	➢ Vaccine safety and rumor control (67%)
	➢ Vaccine distribution-including both quantities and timing of distribution (17%)
	➢ Vaccine efficacy (11%)
Difficulty in managing the helpline (14%)	➢ Lack of staff available to cover the service (31%)
	➢ Lack of medical expertise (19%)
	➢ Inadequate publicity of the helpline number (19%)
	➢ Lack of a triage system to re-direct calls (especially those with specific medical content) (12%)
	➢ Lack of clarity of when to activate and de-activate the service (12%)
Obstacles in the process of releasing timing information (12%)	➢ Agencies’ ability to process and release new information was complex and time consuming (50%)
Difficulty in developing appropriate client advertising of flu clinics (9%)	➢Difficulty in predicting the timing and availability of vaccine and consequent frequent changes in the flu clinics’ schedules (45%)
Inconsistency of messages (8%)	No subtheme identified
Lack of activation of a Joint Information Center (JIC) (8%)	No subtheme identified

* Number of statements referring to a specific theme over the number of statements identified for the selected capability. **Number of statements referring to specific subtheme over the number of statements identified for the selected subtheme.

**Table 2:** Analysis of themes and sub-themes for the capability of information sharing.

Information Sharing ( *81 statements*)	
Most frequent themes ( *proportion* *)	Most frequent subthemes ( *proportion* **)
Difficulty in sharing information with external partners (31%)	➢ Lack of communication with health care providers and schools (39%)
	➢ Differences in terminology used by different organizations (<5%)
	➢ Differing communication systems (<5%)
	➢ Confusion about the role of public health within the response system (<5%)
	➢ The existence of multiple, competing channels of communication (<5%)
	➢ Inconsistency of messages (<5%)
Lack of training in the use of technology (27%)	➢ Lack of training in the use of specific communication systems for example, the Health Alert Network (HAN) program and Web-EOC (14%)
	➢ Problems with the use of more generic instruments of communication such as conference calling, radio systems and appropriate website design to facilitate dissemination of information across agencies (13%)
Difficulty in tracking information (25%)	➢ Information overload and redundancy (29%)
	➢ Excessively frequent changes in the information being produced (18%)
	➢ Very lengthy situation reports (12%)
Difficulty in sharing information across different groups within the same organization (15%)	➢ Lack of clarity between daily job *versus* emergency operations priorities (28%)
	➢ Unnecessary duplication of efforts and unclear staff notifications (<5%)

* Number of statements referring to a specific theme over the number of statements identified for the selected capability. **Number of statements referring to specific subtheme over the number of statements identified for the selected subtheme.

### 4.2. Information Sharing

Out of the thirty-one AARs describing a specific challenge associated with the capability of information sharing, we were able to extrapolate eighty-one statements, of which fifty-eight were derived from AARs focusing on the response to H1N1, and twenty-three from AARs describing the response to a hurricane (eighteen on Gustav and/or Ike and five on Katrina). Content analysis of the eighty-one statements was performed and eleven themes and fifty-two sub-themes were derived. Among the eleven themes, the three most frequently reported were: difficulty in sharing information with external partners (31%), lack of training in the use of technology (27%), and difficulty in tracking information (25%).. 

Analysis of subthemes helped us to describe specific issues within each of the challenges reported above. In terms of communication with external partners, a main issue was a lack of communication with health care providers and schools (39%) as a result of poor organizational and individual level connections among agencies. One specific example cited in an AAR by one organization was that, during the H1N1 pandemic, they reported that staff had no access to a comprehensive list of healthcare providers that they felt should be included in the information sharing process. In terms of lack of training in the use of technology, two main subthemes emerged. The first was related to lack of training in the use of specific communication systems (14%), such as the Health Alert Network (HAN) program and the Web-EOC. The second in frequency (13%) was related to lack of training in the use of more generic instruments of communication such as conference calling (lack of knowledge in the use of conference calls programs and lack of clarity on how to structure the conference calls), radio systems (device programming errors, issues related to lack of wireless system reliability), and challenges in developing websites appropriately designed to facilitate dissemination of information across agencies. Tracking of information (*i.e.*, tracing and capturing information from multiple sources) was also cited as a challenge because of information overload and redundancy (29%), excessively frequent changes in the information being produced (18%), and very lengthy situation reports (12%). A list of most frequent themes and subthemes derived from the analysis of this capability is provided in Table 2. 

### 4.3. Emergency Operations Coordination (EOC)

Out of the thirty-two AARs describing the capability of EOC operations, we were able to extrapolate one-hundred-seventy-nine statements describing specific challenges, of which one hundred-nineteen were derived from AARs focusing on the response to H1N1 and sixty from AARs describing the response to hurricanes. Content analysis of the one-hundred-seventy-nine statements was performed, and fifteen themes and fifty-six sub-themes were derived. Among the fifteen themes, the three most frequently reported were: (1) confusion in roles and responsibilities across responders assigned to the EOC (23%); (2) lack of use of the Incident Command System (ICS) (17%), and (3) difficulty in consistently developing and using situation reports (14%). 

Some of the subthemes that emerged in terms of *confusion in roles and responsibilities* in the EOC included confusion of job tasks and functions among various response personnel (43%), poor depth of knowledge of individual response roles among response personnel (19%), overlap in roles and responsibilities among various sections of the EOC (14%), use of new staff for supervisory roles (10%), and switching of roles without coordination (7%). Lack of use of the ICS among responders was very commonly cited as due to lack of training (68%). In terms of *difficulty in developing situation reports*, subthemes included difficulty in gathering data resulting in fragmented and inconsistent reports (46%), lack of an organized format making the reports difficult to read (12%), insufficient distribution of reports to appropriate parties (12%), lack of follow-up on the information reported (12%), and lack of familiarity with electronic tracking systems (12%). A list of most frequent themes and subthemes derived from the analysis of this capability is provided in Table 3. 

**Table 3:** Analysis of themes and sub-themes for the capability of emergency operation coordination (EOC).

Emergency Operations Coordination (EOC) ( *179 statements*)	
Most frequent themes ( proportion *)	Most frequent subthemes ( *proportion* **)
Confusion in roles and responsibilities within ICS (23%)	➢ Confusion of job tasks and function among various response personnel (43%),
	➢ Poor depth of knowledge of individual response roles among response personnel (19%)
	➢ Overlap in roles and responsibilities among various sections of the EOC (14%)
	➢ Use of new staff for supervisory roles (10%)
	➢ Switching of roles without coordination (7%)
Poor familiarization and/or use of the Incident Command System (17%)	➢ Training needs including leadership training (68%)
	➢ Systems’ lack of formal command structure (<5%)
	➢ Lack of compliance and competence in filling out the required ICS forms (<5%)
Difficulty in aggregating and utilizing situation reports (14%)	➢ Difficulty in gathering and collecting data resulting in fragmented and inconsistent reports (46%)
	➢ Lack of organized format that made the reports difficult to read (12%)
	➢ Insufficient dissemination of reports to appropriate entities (12%)
	➢ Lack of follow-up on information reported (12%)
	➢ Lack of familiarity with electronic tracking systems (12%)
Communication and coordination issues (10%)	➢ Poor communication among various sections of the EOC (32%)
	➢ Poor communication and coordination between the EOC and external partners (32%)
	➢ Poor and inconsistent coordination between the EOC and the media (21%)
Confusion in response activities *versus* day to day activities (5%)	➢ Failure to release responders from daily activities (44%)
	➢ Lack of clear assignment of response roles among responding personnel (33%)
Poor incident action planning (5%)	➢ Difficulty in distributing incident action plans (33%)
	➢ Difficulty in developing incident action plans (22%)
	➢ Lack of clearly defined response objectives (22%)
	Lack of implementation of a developed incident action plan (11%)

* Number of statements referring to a specific theme over the number of statements identified for the selected capability. **Number of statements referring to specific subtheme over the number of statements identified for the selected subtheme.

### 4.4. Distribution of Themes and Subthemes across Types of Incidents

For the capabilities of information sharing and EOC operations, we could tabulate frequencies of themes and subthemes by type of event: H1N1 pandemic *versus* hurricanes (Table 4). However, for the capability of emergency public information and warning such analysis could not be performed due to the fact that 96% of statements were derived from the response to H1N1, and essentially very little information was provided in the AARs about communication to the public during the response to hurricanes. 

**Table 4:** Distribution of themes across types of incidence.

Top Recurring challenges for EOC operations by event	Total n = 179	H1N1 (n = 119)	Hurricanes (n = 60)	*P-*value ^a^
Confusion in roles and responsibilities	42 (23%)	31 (26%)	11 (18%)	0.5
Poor familiarization and/or use of the Incident Command System	31 (17%)	16 (14%)	15 (25%)	0.2
Difficulty in aggregating and utilizing situation reports	26 (14%)	19 (16%)	7 (12%)	0.6
Communication and coordination issues among various sections of the EOC and/or with external partners	19 (11%)	11 (10%)	8 (13%)	0.5
**Top Recurring challenges for Information sharing by event**	**Total n = 81**	**H1N1 (n = 58)**	**Hurricanes (n = 23)**	***P-*value^a^**
Communication with external partners	25 (31%)	23 (40%)	2 (9%)	0.06
Information tracking	20 (25%)	13 (22%)	7 (30%)	0.6
Use of technology	22 (27.5%)	12 (21%)	10 (43%)	0.2
Communication across different teams within the same organization	12 (15%)	11 (19%)	1 (4.3%) ^b^	0.2

^a ^*P-*value calculated using Fisher’s exact test. ^b ^Not a top recurring challenge for hurricanes.

Results showed that, for the capability of *information sharing,* similar themes were identified regardless of the type of incident. During H1N1, the top three challenges were: (1) communication with external partners (40%); (2) information tracking (22%); and (3) communication across different teams within the same organization (19%). The top three challenges in the response to hurricanes were: (1) use of technology (43%); (2) information tracking (30%); and (3) communication with external partners (9%). Despite similarities in the challenges being reported, specific response issues were slightly different between the two types of incidents. In terms of communication with external partners during H1N1, the main challenge for the public health agencies was to establish an effective communication with new partners such as the private sector, healthcare organizations and school districts. During the response to hurricanes, a frequent challenge was inconsistency in the use of terminology across agencies causing misunderstanding of the content of the messages. Information tracking was a challenge for both the response to H1N1 and to hurricanes with cases ranging from redundancy of information to lack of information. During the response to hurricanes, the damages to the communication infrastructure such as radio and wireless communications systems were the cause of major communication difficulties. Lack of training in the use of Health and Homeland Alert Network (HHAN) and Web-EOC was found to be a challenge both during H1N1 as well as during the response to hurricanes. Finally, communication across different teams within the same organization seemed to be a greater issue during H1N1 than during the response to hurricanes due to lack of clarity about shifts between routine activities and emergency operations. This may have been, at least in part, due to the extended timeframe of the response to H1N1.

The major themes noted in the AARs discussing EOC operations were also similar for the two different types of events being studied, though not in similar order. The top three themes that emerged in the H1N1 response were: (1) confusion in roles and responsibilities (26%); (2) difficulty in developing and using situation reports (16%); and (3) lack of use of the ICS (13%). The top three themes that emerged from the response to hurricanes were: (1) confusion in roles and responsibilities (18%); (2) lack of use of the ICS (25%), and (3) communication and coordination issues among various sections of the EOC and/or with external partners (13%). Examples cited in AARs include EOC personnel reporting to multiple people causing diversion of information from its intended flow, and lack of clarity about each agency’s primary designated contacts at each partner organization. 

### 4.5. Notes on the Structure of the AARs

During the content analysis of the ninety-one AARs selected for this study, we also took note of each AAR’s structure and format as well as the way authors defined and examined “capabilities”. Among the AARs that had descriptions of performance on at least one of the three capabilities being examined in our study, only 67% had a report structure with text specifically referring to the concept of a “capability.” Of those, approximately 80% of those reports followed the Target Capability List (TCL) [13] definition to structure the report. In addition, there was no consistency on what was included in each capability section, on how the capabilities were named or defined, or on the “response issues or functions” to be included within each capability and incident. As an example, surprisingly, over 90% of AARs describing the response to a hurricane had no observations on the capability of emergency public information and warning.

## 5. Discussion

Knowledge management is a discipline that attempts to use a variety of strategies and practices to identify, describe and disseminate the insights and experiences gained by individuals and groups over time [3,14].When these insights and experiences are gathered together in documents such as AARs and critically analyzed, they comprise an opportunity to identify common and/or recurring systems-level challenges. If the identified insights and experiences have recurring themes across different types of threats and across multiple types of systems, they also present a direct mandate for responders, organizations, and systems to address such challenges and create a requirement to test them in future exercises to ensure that planned improvements are successful. In other words, evidence of common and recurring challenges must be directly addressed by planners, and any implemented improvements must be tested through the iterative cycle of planning, testing, measuring and improving, as supported in the FEMA guidance for the development of AARs and Improvement Plans (IPs).

The LLIS.gov system is an example of a repository of information where insights and experiences that could support national knowledge management are documented following the response to large scale emergencies. While the existence of this repository is well known to public health and emergency management agencies, as evidenced by the number of AARs continuously uploaded to the system, no research to date had focused on the possible use of the database for knowledge management purposes. In our study, we have identified a number of common themes and subthemes of “lessons learned” that we were able to derive from the AARs submitted to the LLIS system that are representing cross-cutting lessons. We believe many of these themes identified through content analysis provide data in support of several current national and international initiatives to improve preparedness and response efforts in the areas of communications systems and situational awareness, use of the ICS, information sharing and public information and warning. Further, we believe that recurring content analysis of AARs databases has substantial potential utility for local and state exercise planners. Currently, creation of a multi-year training and exercise program (MYTEP), as recommended in the HSEEP, is a complicated endeavor that relies on many factors including state and/or local capabilities assessments, homeland security strategy, and past local AARs and IPs. The AARs and IPs most commonly used for MYTEPs, however, are limited by the exercises and incidents that have recently occurred in specific local realities. Instead, if the most common response challenges that have routinely proved problematic could be identified within nationally aggregated pools of AARs, such data could be used by practitioners when drafting their MYTEP, so that testing of plans and response to such challenges could be included, and hopefully not to be “learned” again. 

A limitation of our study is the fact that only two types of incidents were taken into consideration: the H1N1 pandemic and the response to hurricanes. Indeed, other emergency incidents such as floods, mass shooting, chemical spills, radiological events were not included in our study, however, at the time of the data extraction, the database contained approximately 400 AARs from public health and healthcare entities analyzing their response to real events. Our analysis, therefore, included nearly one quarter of the total number of public health AARs available, which we believe is a fairly reasonable size upon which to draw preliminary conclusions. 

As recommended by the HSEEP guidelines, some AARs were noted to include recommendations for improvements that would address the problems they described. However, in many cases, the recommendations were often rather generic and could not be translated into concrete actions. For example, one agency reported the need to “*create a robust* *crisis emergency response communication plan*,” but was unable to describe further what such a plan would entail. Another agency suggested that, when communicating, “*the information should be given in an effective way in order to reach the target population*,” leaving the reviewers with questions about the definitions of “effective” and “target audience?

Many of the AARs that we examined also lacked specific examples to support the individual authors’ statements about what went wrong and lacked root cause analysis of the response challenges experienced. Further, the frequent lack of use of a consistent structure in AARs makes identification of specific problems and root causes extremely challenging and time consuming when trying to aggregate lessons learned. Overall, we believe that providing both more detailed examples of response challenges, as well as greater use of root cause analysis methodology within the AARs could help broaden others’ ability to understand the problems documented in an AAR and improve their ability to apply the lessons learned to their own entity and system. Further, we believe that improved use of a consistent structure for all AARs entered in the system would facilitate faster and simpler use of structured approaches to identify the problems in the response that may be common to multiple responders and therefore need urgent attention by all. At present, individual state and local health departments, as well as healthcare providers, commonly produce AARs of widely ranging structure and quality following emergency and disaster events. This is likely due to a number of internal factors, including differences among entities in expertise in event evaluation, limited personnel availability, competing priorities, political concerns, and others. It is also likely due, to external factors as well, such as the competing and conflicting federal capabilities lists, cumbersome current AAR guidance and structures, and concerns about the use of AAR data at the state and federal levels for accountability purposes. 

We believe there are possible actions that might improve the overall quality of AARs that may lead to higher value data being reported and improved identification of systematic response challenges. First, the federal government might make available examples of “best practice” of AARs that could guide authors who are attempting to improve their documents. Second, national AAR repositories could be indexed and be searchable by public health and health care emergency preparedness capabilities to facilitate the identification of common response challenges and add value and meaning in the use of the capability-based approach when drafting AARs. Third, the government could consider building financial incentives that encourage meeting certain quality criteria in the production of AARs, such as use of the capabilities frameworks, performing root cause analysis, and providing concrete examples, though we acknowledge that, in the current fiscal environment this might be difficult, and the magnitude of the incentive would likely not be substantial enough to motivate change. Nonetheless, successfully encouraging local and state practitioners to improve the overall quality of the AARs produced and submitted to central repositories, will have greater potential for learning not just for the individual entities crafting the AARs, but for the health and public health sector as a whole. 

## 6. Conclusions

By performing a systematic analysis of ARRs submitted through the LLIS.gov system, we were able to identify common challenges that have consistently emerged during the public health responses to different types of incidents. These challenges represent lessons that do not need to be “learned again” in future disasters, but rather problems that must be faced now in the planning, testing, measuring, and implementing cycle of public health and emergency management (see Table 1, Table 2 and Table 3). By reviewing the AARs, we believe that public health and emergency management leaders can develop data-driven support system for identifying key areas of concern when prioritizing planning efforts. Although knowledge management approaches are known to work when all members of the target community have the same technical goals, are motivated by a common interest, are organized on a flat hierarchy, and are receptive to innovation [15]. Further, we believe that future exercises should include these challenges in their objectives as a mechanism for ensuring that testing and planning efforts are successful. Our study suggests the value of AARs as potential tools to learn from real incidents and underlines the need to develop systematic ways to synthesize the lessons learned from such documents. 
